# Bilateral Curvularia Keratitis

**DOI:** 10.18502/jovr.v15i4.7796

**Published:** 2020-10-25

**Authors:** Ajit Kumar, Ashi Khurana

**Affiliations:** ^1^C L Gupta Eye Institute, Ram Ganga Vihar, Moradabad, India

##  PRESENTATION

A 45-year-old female presented with bilateral ocular pain and redness. The best-corrected visual acuity (BCVA) at presentation was 20/30 OD and 20/100 OS. Slit lamp examination revealed 2×2 mm epithelial defects with mid-stromal infiltrate and dark brown corneal plaques on both eyes. The plaque surface was dry and raised with brownish pigmentation. The margins of the stromal infiltrate had feathery edges. Corneal scraping was performed bilaterally. The Sabouraud's dextrose agar culture of each eye grew woolly, gray to black colonies by the seventh day. A *Curvularia* sp. was identified. The patient was treated with intensive medical treatment, including topical natamycin suspension (5%) applied every half hour OU for five days along with cycloplegics. Antifungal therapy was decreased to every 2 hours for seven days. Response to medical treatment alone was favorable, with significant improvement in BCVA. A 12-day follow-up examination revealed a faint paracentral scar in the right eye and central corneal opacity in the left eye, but both ulcers had resolved completely. The final BCVA upon follow-up was 20/20 OD and 20/80 OS.

**Figure 1:**
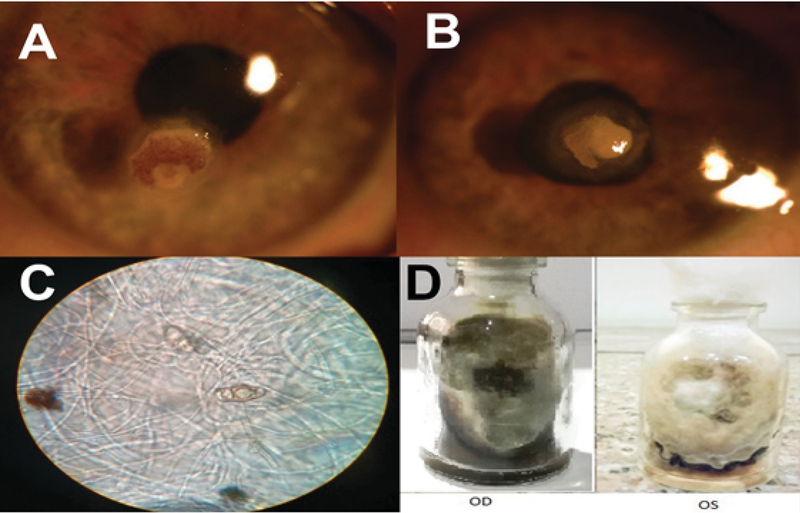
Bilateral Curvularia Keratitis. (A & B): Slit lamp photograph of right and left eye in diffuse elimination showing 2 × 2 mm of epithelial defect with overlying pigmanted plague. (C): 10% KOH direct mount preparation from plate of both eyes (40×) showing pigmented multiple cell conidia. (D) SDA siant culture showing woolly greyish black colonies.

##  DISCUSSION

There has been increased incidence of keratomycosis due to pigmented fungi.^[1]^ In our patient, there was no history of preceding trauma, agricultural work, allergic conjunctivitis, ocular surgery, use of contact lenses or any other systemic diseases. Topical natamycin suspension (5%) was used as the first line medication as it has excellent efficacy *(in vitro)* against pigmented fungi. It may also enhance the therapeutic effect of voriconazole. In this case, ulcers of both eyes resolved without complications.

Reports on bilateral fungal keratitis are rare. This patient was considered immunocompetent on the basis of her behavioral pattern, while most reports include people with compounding disease. Prajana et al reported simultaneous bilateral fungal keratitis caused by *Aspergillus flavus* and *Curvularia lunata *in a patient with lamellar ichthyosis.^[2]^ Labiris et al discovered bilateral *Fusarium oxysporum* keratitis associated with laser in situ keratomileusis.^[3]^ Zhou et al reported bilateral *Rhizopus* keratitis in a cocaine user.^[4]^ Chaniyara et al also reported a case of bilateral fungal keratitis.^[5]^ Although *Curvularia sp*. has been implicated as a cause of fungal keratitis, concurrent bilateral occurrence is unique.

## References

[1] Chowdhary A, Singh K. Spectrum of fungal keratitis in North India. *Cornea* 2005;24:8–15. 10.1097/01.ico.0000126435.25751.20 15604861

[2] Prajna NV, Rao RA, Mathen MM, Prajna L, George C, Srinivasan M. Simultaneous bilateral fungal keratitis caused by different fungi. *Indian J Ophthalmol* 2002;50:213. 12355697

[3] Labiris G, Troeber L, Gatzioufas Z, Stavridis E, Seitz B. Bilateral Fusariumoxysporum keratitis after laser in situ keratomileusis. *J Cataract Refract Surg* 2012;38:2040–2044. 10.1016/j.jcrs.2012.08.037 23079318

[4] Zhou M, Farooq AV, Andreoli MT, Ali M, Traish AS. Bilateral Rhizopus keratitis in a cocaine user. *Can J Ophthalmol* 2016;51:e21–e23. 10.1016/j.jcjo.2015.09.018 26874166

[5] Chaniyara MH, Pujari A, Urkude J, Sharma N. Bilateral fungal keratitis with ring infiltrates: a rare scenario. *BMJ Case Rep* 2017;2017:bcr-2017. 10.1136/bcr-2017-221226 PMC5652377 28993355

